# Prognostic and Predictive Values of Subcellular Localisation of RET in Renal Clear-Cell Carcinoma

**DOI:** 10.1155/2016/6870470

**Published:** 2016-03-22

**Authors:** Lei Wang, Yu Zhang, Yu Gao, Yang Fan, Luyao Chen, Kan Liu, Qingyu Meng, Chaofei Zhao, Xin Ma

**Affiliations:** Department of Urology, State Key Laboratory of Kidney Diseases, Chinese PLA General Hospital, Chinese PLA Medical Academy, Beijing 100853, China

## Abstract

Metastatic renal cell carcinoma (RCC) presents a poor prognosis and an unpredictable course. To date, no validated biomarkers can predict the outcome of RCC. Ongoing efforts are conducted to identify the molecular markers of RCC progression, as well as the targets for novel therapeutic approaches. RET is a tyrosine kinase receptor which has been investigated as a possible target in other cancers because it is involved in oncogenic activation. To evaluate the predictive and prognostic functions of RET in ccRCC, a tissue microarray study was conducted on 273 ccRCC patients. Results showed that both RET cytoplasmic and nuclear expression were independently associated with PFS and OS, and the combined RET cytoplasmic and nuclear statuses demonstrated that the ratio of high nuclear RET and cytoplasmic RET was the strongest predictor of both PFS and OS. The high cytoplasmic RET expression retained its independent poor prognostic value in targeted drug treated patients. The RET nuclear expression was associated with distant metastasis. Moreover, the RET nuclear expression was an independent predictor of ccRCC postoperative metastasis. In conclusion, RET may be useful in prognostication and can be used at initial diagnosis to identify patients with high potential to develop metastasis.

## 1. Introduction

Renal cell carcinoma (RCC) represents 2% to 3% of all cancers. In the past two decades, an annual increase of approximately 2% was found in RCC incidence worldwide [1]. One-third of patients diagnosed with kidney cancer presented evidence of metastatic disease at the time of diagnosis, and up to half of those treated for localised disease showed eventual relapse [2]. Current treatment regimens for metastatic RCC achieve modest response rates, and relatively few patients become long-term survivors. The length of response and survival benefit of therapy varies considerably among patients. Hence, novel approaches to prognostication and therapy of metastatic diseases are urgently needed.

Recent achievements in basic sciences have increased the understanding about the molecular pathways underlying different RCC subtypes. Multiple factors have established benefits from the prognostic function of RCC. The natural history of RCC is complex and is influenced by factors other than the disease stage [3]. Therefore, understanding how the complex interactions among multiple prognostic factors contribute to the clinical behaviour of RCC is essential for patient assessment, outcome prediction, and therapy planning.

The glial-cell-line-derived neurotrophic factor receptor (RET) is a tyrosine kinase receptor which transduces signals for cell growth and differentiation. RET can undergo oncogenic activation in vivo and in vitro through cytogenetic rearrangement [4–7]. This kinase is also often found to be abnormally activated in the thyroid, generally through sporadic and inherited gene mutations. Thus, both the oncogene RET and the RET receptor tyrosine kinase may contribute to cancer development [8]. The oncogenic activation of the RET gene is recognised as an early pathogenic event in cancers, and this occurrence subsequently induces the downstream signalling events involving the MEK/ERK-, PI3K/AKT-, and phospholipase C*γ*- (PLC*γ*-) dependent pathways [9]. The prognostic function of RET has been found in various cancers. Although no selective RET inhibitors have yet been developed for clinical use, few commercially available multikinase inhibitors, such as sorafenib and sunitinib, have demonstrated activity against the RET kinase. Despite the unquestioned role of RET as an important regulator of tumour pathophysiology, very few attempts have explored its prognostic role in RCC [10, 11].

The most common type of RCC is renal clear-cell carcinoma. Our previous study in the laboratory showed that RET is an independent prognosticator in patients with metastatic clear-cell RCC (ccRCC). On the basis of our previous data, we propose RET as a novel prognostic marker in ccRCC, which may also be useful to determine specific therapeutic approaches for various patients. As such, this topic is worthy of further investigation.

## 2. Materials and Methods

### 2.1. Patients

Our study consisted of 273 patients who underwent nephrectomy and had a pathologically confirmed diagnosis of RCC with a component of clear-cell histology. These patients were treated at the PLA General Hospital from January 2006 to December 2012. The diagnosis of metastatic disease was determined at initial presentation. Among the patients, 158 had distant metastasis, and 107 received VEGFR-targeted tyrosine kinase inhibitors (sorafenib or sunitinib). The median age was 55 years (18 years to 81 years). A total of 204 were males, and 69 were females. The follow-up database was closed in July 2015, after a median follow-up of 54.1 months (27 months to 102 months, IQR: 42 months to 69 months). The median PFS was 16.7 months (1 month to >102 months, 6.7 months to >50 months), and the median OS was >34 months (1 month to >102 months, 18 months to >54 months). A total of 112 patients (41%) were alive at the time of analysis. For each patient, the following clinicopathological data were collected: age, sex, T stage, nuclear grade, and ECOG performance status. Written informed consent for a tumour-oriented study was obtained from each patient prior to sample collection. The study was approved by the Protection of Human Subjects Committee of the Chinese People's Liberation Army General Hospital. This research was conducted in accordance with the Declaration of Helsinki.

### 2.2. Tissue Array Construction

Formalin-fixed paraffin-embedded primary tumour specimens were obtained from the Department of Pathology at the PLA General Hospital. Three core-tissue biopsies with 1.0 mm diameter were collected from the selected morphologically representative regions of each paraffin-embedded RCC and precisely arrayed using a custom-built instrument (Quick-Ray UT-06, UNITMA). Sections of the resulting tumour tissue microarray block with 4 *μ*m thickness were transferred to glass slides by using the paraffin sectioning aid system to support the cohesion of 1.0 mm array elements.

### 2.3. Immunohistochemistry

The sections were heated at 56°C for 30 min, deparaffinized with xylene and rehydrated with a descending series of ethanol. For antigen repair, sections were dipped in citric acid buffer at 95°C for 20 min, cooled down to room temperature, incubated with 3% H_2_O_2_ in methanol for 30 min at 37°C, and then blocked with normal goat antiserum for 30 min at 37°C. Afterwards, the sections were incubated with the indicated primary antibodies overnight at 4°C in accordance with the standard procedures. The primary antibodies include monoclonal RET (Abgent, San Diego, CA) (1 : 100 dilution). After washing the sections in PBS, they were treated with biotin-labelled serum (1 : 200) for 30 min, rinsed with PBS, and then visualised using an Envision kit/HRP (DAB) (ZSGB-BIO, China) (DAB). Retrograde alcohol dealing was then performed. The sections were counterstained in haematoxylin, mounted in Permount, and then evaluated microscopically. Negative control was performed by replacing the primary antibody with goat serum.

The evaluation of expression involved subcellular localisation (nucleus and cytoplasm). The immunostaining level was determined by counting 1,000 cells in 10 large graticules visible in the microscope. The results were semiquantitatively reported on a scale from 0 to 3 for intensity, where 0 was negative, 1 was weak, 2 was moderate, and 3 was strong. The percentage of tumour staining was reported as 0% to 100% in increments of 10%. A composite score was formed using the product of the intensity and percentage of tumour cell cytoplasmic or nuclear staining. All slides were examined and scored independently by three pathological consultants without knowing the patients' clinical data. For any disagreement, a consensus was reached by discussion. Measurements of three cores per sample were averaged for the analysis. The overall score used for subsequent statistical analysis was the pooled mean from the three spots of the same tumour.

### 2.4. Statistical Analysis

We defined progression-free survival (PFS) as the time between the date of nephrectomy and the date of radiological progressive disease (PD), clear clinical evidence of PD, or death. Patients who had not progressed at database closure were censored during the final follow-up. If the PD date was unknown, we censored the PFS at the last tumour assessment. Metastasis-free survival (MFS) was defined as the time between the date of nephrectomy and the date of distant metastasis or death. Overall survival (OS) was defined as the time between the date of nephrectomy and the date of death or the last date of follow-up.

The primary study end-point was the association of RET expression with the clinicopathological features and prognosis. Continuous variables were reported as medians [range, interquartile range (IQR)]. All proteins investigated as well as the age and Fuhrman grade were not normally distributed by Kolmogorov–Smirnov test. To study the relationships between the covariates, Spearman's Rho test was used for comparisons of bivariate and categorical variables. The covariates were examined using a logistical regression model. The multivariable associations of the clinicopathological variables with survival outcome were examined through Cox regression analyses. Multivariable analysis included age, gender, T stage, tumour metastasis status, Fuhrman grade, presence of targeted drug treatment, and ECOG PS as covariates (clinical factors associated with *p* < 0.05 with a specific variable were used as covariates for that specific variable) [12]. RET subcellular immunostaining associated with survival outcome in the multivariable analysis was further analysed using the Kaplan–Meier method and compared using the log-rank test. Immunostaining was dichotomised according to median cut-off. A two-sided *p* < 0.05 was considered significant in all stages of this analysis. All the statistical analyses were conducted using SPSS version 19.0 and GraphPad 6.0.

## 3. Results

### 3.1. Immunohistochemistry

**Figure 1:**
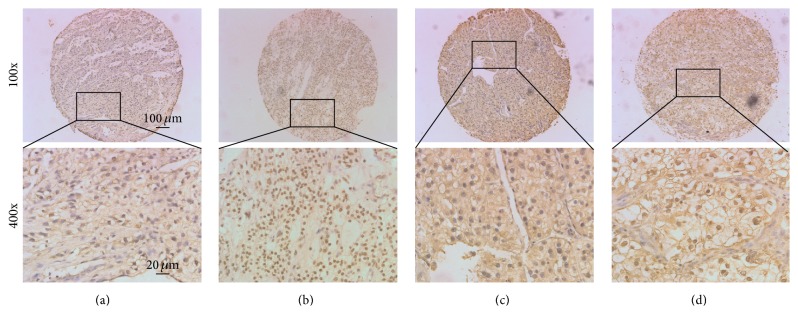
Expression of nuclear and cytoplasmic RET. Shown are representative figures of low RET nuclear and cytoplasmic (panels (a)), high RET nuclear only (panels (b)), high RET cytoplasmic only (panels (c)), and high RET nuclear and cytoplasmic (panels (d)) expression in ccRCC.

Immunohistochemistry for RET expression was optimised in a set of eight archived whole-tissue blocks. Immunoreactivity was widely divergent among different tumour samples, but the staining levels/percentages of nuclei were consistent throughout a given tumour (Figure 1, data not shown). We surmised that these features made RET immunohistochemistry appropriate for tissue microarray analysis. The median tumour cell RET cytoplasmic and nuclear immunostaining in all ccRCC were 110% and 80%, respectively. Specimens with expression of >110% RET cytoplasm were categorised as high RET cytoplasmic expression tumours, and specimens with expression of >80% RET nucleus were categorised as high RET nuclear expression tumours.

### 3.2. Association of RET Expression with Survival Outcome

**Figure 2:**
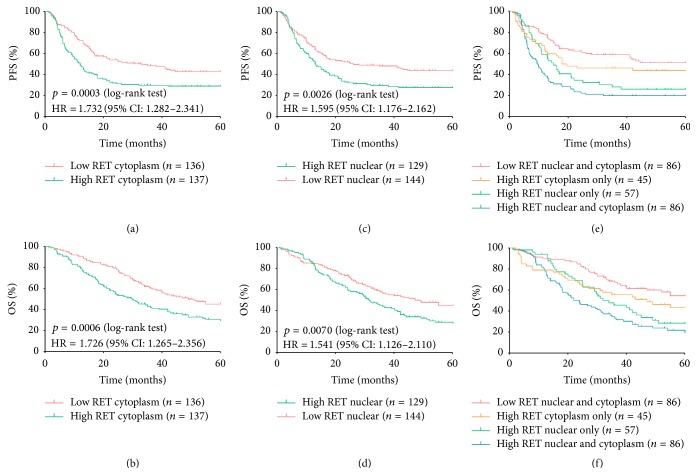
Association of RET subcellular expression with PFS and OS. (a) Patients with high RET cytoplasmic expression had significantly worse PFS; (b) patients with high RET cytoplasmic expression had significantly worse OS; (c) patients with high RET nuclear expression had significantly worse PFS; (d) patients with high RET nuclear expression had significantly worse OS; (e) patients with high RET nuclear and cytoplasmic expression had the worst PFS of all patients; (f) patients with high RET nuclear and cytoplasmic expression had the worst OS of all patients. Log-rank test was used to study the differences between the covariates.

The clinicopathological features and survival outcomes were compared by the Spearman's Rho test. Both RET cytoplasmic and nuclear immunostaining were associated with poor survival outcomes. Patients with high RET cytoplasmic expression displayed a shorter median PFS and OS than those with low RET cytoplasmic expression. Tumours with versus without high RET nuclear expression presented similar shorter median PFS and median OS, but more frequent distant metastasis (*p* values are listed in Table 1). The expression of cytoplasmic RET was associated with shorter PFS (HR = 1.732, 95% CI: 1.282 to 2.341, *p* = 0.0003) (Figure 2(a)) and shorter OS (HR = 1.726, 95% CI: 1.265 to 2.356, *p* = 0.0006) (Figure 2(b)). The high RET expression in the nucleus was associated with shorter PFS (HR = 1.575, 95% CI: 1.176 to 2.162, *p* = 0.0026) (Figure 2(c)) and shorter OS (HR = 1.541, 95% CI: 1.126 to 2.110, *p* = 0.0070) (Figure 2(d)).

The complete multivariable Cox regression analyses are summarized in Table 2. The association between nuclear RET expression and OS lost significance when analysed in a dichotomised manner (*p* = 0.307). Therefore, we included continuous fashion in multivariable analysis, and both cytoplasmic and nuclear RET expression became independently associated with PFS and OS when analysed continuously (HR and *p* values are listed in Table 2). Multivariable Cox regression models showed that high Fuhrman grade, ECOG PS, T stage, and distant metastasis were independently associated with shorter PFS and OS, whereas targeted treatment was independently associated with longer PFS and OS. Obviously, distant metastasis was the strongest predictor of both PFS and OS (with the largest hazard ratio). The combined RET nuclear and RET cytoplasmic statuses demonstrated that the high RET nuclear and RET cytoplasmic expression were the strongest predictor of both PFS (2.466, 1.639 to 3.711, *p* < 0.001) and OS (2.294, 1.499 to 3.510, *p* < 0.001) (Table 2(E), Figures 2(e) and 2(f)).

**Figure 3:**
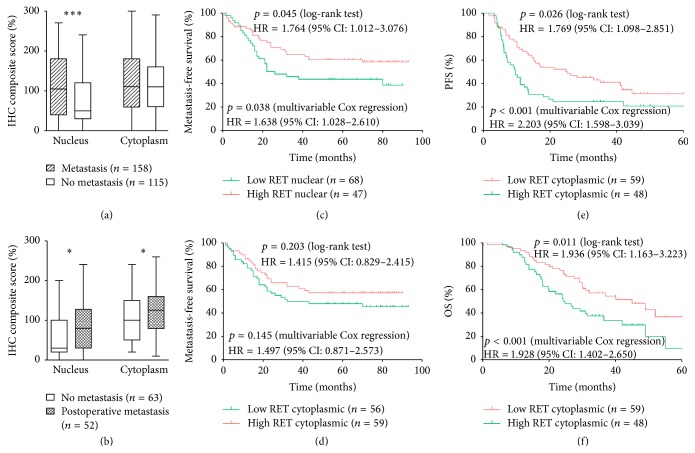
Association of RET expression with metastasis and prognostic function of RET in targeted drug treated patients. (a) Expression of nuclear and cytoplasmic RET in no metastatic tumours and metastatic tumours; (b) expression of nuclear and cytoplasmic RET in no postoperative metastatic tumours and postoperative metastatic tumours; unpaired *t*-test was used to study the relationships between the covariates, ^*∗*^ *p* < 0.05, and ^*∗∗∗*^ *p* < 0.0001; (c) patients with high RET nuclear expression had significantly shorter MFS; (d) patients with high RET cytoplasmic expression had shorter median MFS, but *p* value was not significant; (e) patients with high RET cytoplasmic expression had significantly worse PFS; (f) patients with high RET cytoplasmic expression had significantly worse OS.

A total of 107 patients received VEGFR-targeted tyrosine kinase inhibitors (sorafenib or sunitinib). Both sorafenib and sunitinib demonstrated activity against the RET kinase. Therefore, we investigated whether RET retained its independent prognostic value in patients who received targeted drugs. Multivariable Cox regression and Kaplan-Meier methods were adopted in further analyses because the cytoplasmic expression was a strong predictor in the above tests. As a result, cytoplasmic RET expression was associated with shorter PFS (1.769, 1.098 to 2.851, *p* = 0.026) and OS (1.936, 1.163 to 3.223, *p* = 0.011). Moreover, the cytoplasmic RET expression was an independent predictor of PFS (2.203, 1.598 to 3.039, *p* < 0.001) and OS (1.928, 1.402 to 2.650, *p* < 0.001) in metastatic ccRCC (Figures 3(e) and 3(f)).

### 3.3. Association of RET Nuclear Expression with Distant Metastasis

Unpaired *t*-test showed that tissues sampled from tumours with distant metastasis presented higher RET nuclear expression than that of samples from tumours without distant metastasis (Figure 3(a)). A multivariate logistic regression model was employed to validate the association between RET nuclear expression and distant metastasis. The RET nuclear expression was selected as a rank variable, and the tumour metastasis status (presence or absence of distant metastasis) was selected as a dependent variable. Other dependent variables include age, gender, T stage, Fuhrman grade, and ECOG PS as covariates. The results showed that tumours with versus without high RET nuclear expression presented more frequent distant metastasis (HR = 2.262, 95% CI: 1.527 to 3.352, *p* < 0.001). This analysis revealed that the RET nuclear expression was an independent predictor of ccRCC distant metastasis.

### 3.4. RET Nuclear Expression as an Independent Predictor of Postoperative Metastasis

To link the RET expression to the postoperative metastasis risk, 115 patients without metastases were  included in the analysis. A total of 52 patients (46%) developed metastases or died at the end of the follow-up. Unpaired *t*-test showed that tissues sampled from tumours with postoperative metastasis achieved both higher RET nuclear and cytoplasmic expression than samples from tumours without postoperative metastasis (Figure 3(b)). The Kaplan–Meier method was employed to validate the association between RET subcellular expression and postoperative metastasis. The results showed that nuclear RET expression was associated with shorter MFS (1.764, 1.012 to 3.076, *p* = 0.045) (Figure 3(c)). The cytoplasmic RET expression presented a worse relative MFS benefit, but the *p* value was not significant (Figure 3(d)). Multivariable Cox regression analyses were performed to validate the independent prognostic value. Immunostaining was selected as continuous variable in Cox regression. The results showed a clear RET expression significantly associated with MFS (1.638, 1.028 to 2.610, *p* = 0.038). Overall, these analyses revealed that the RET nuclear expression was an independent predictor of ccRCC postoperative metastasis.

## 4. Discussion

The natural history of RCC is highly unpredictable. Small renal masses may be accompanied by metastatic disease. Conversely, patients with locally advanced disease may enjoy long-term disease-free survival. Numerous molecular markers, including gene expression profiling and deep and whole-genome-wide sequencing, have been investigated, but none of these techniques have yielded markers or profiles which can improve the predictive accuracy of current prognostic systems [13].

RET can undergo oncogenic activation in vivo and in vitro through cytogenetic rearrangement. The oncogenic activation of the RET gene was detected in various cancers [14, 15]. In the present study, both high cytoplasmic and nuclear immunostaining are associated with poor outcome, whereas high RET nuclear expression is associated with distant metastasis and more frequent postoperative metastasis. In existing reports, the uniform prognostic functions of RET were found in other cancers. For instance, high levels of RET expression in ASCL1+ tumours were associated with significantly shorter OS in stage 1 lung cancer and other lung adenocarcinomas [16]. The RET status was found to correlate with the outcome of sporadic medullary thyroid carcinoma [17–19]. Elevated levels of RET receptors are found in different subtypes of human breast cancers, and high RET correlates with decreased metastasis-free survival [20].

In the present study, the high cytoplasmic RET expression did not only retain its independent poor prognostic value in 107 patients treated with targeted drugs, but it also presented a larger hazard ratio than that in complete patients (HR of PFS: 1.860 versus 2.203, OS: 1.666 versus 1.928). Both sorafenib and sunitinib have demonstrated activity against the RET kinase [10, 11]. Inhibition of the RET receptor tyrosine kinase, which may contribute to cancer development, should improve the outcome of metastatic ccRCC. Patients treated with targeted drugs, who showed low cytoplasmic RET expression, will attain a smaller hazard ratio than that of complete patients. However, the result is inverse in our study. We suppose that targeted drugs can easily block the RET receptor tyrosine kinase and downstream signalling events involving the MEK/ERK and PI3K/AKT pathways in patients with low RET expression. By contrast, the RET receptor tyrosine kinase is difficult to block in patients with high RET expression. We assume that the multikinase inhibitors sorafenib and sunitinib were not active enough against the RET kinase.

In clinical practice, the patient's genetic information is used to lead the treatment decisions of personalised treatment [21]. Spanheimer et al. reported that the combination therapy with antioestrogen and anti-RET in luminal breast cancer exerted a greater effect on cell growth than either therapy alone [22]. In RCC, the multikinase inhibitors are routinely given in advanced-RCC patients and can significantly affect the patient mortality. However, only a subset of patients can be eligible for this treatment. Thus, additional receptor tyrosine kinases (RTKs), which can be useful in RCC therapy, should be developed. Consequently, the RET differences can be exploited in current treatments, as well as in designing future therapeutic options.

Distant metastasis is the main cause of death and therapeutic failure in RCC patients [23]. Although tumour stage, grade, and subtype provide some prognostic information, the metastatic potential of localised RCC is often unpredictable. The present study revealed that high RET nuclear expression is associated with distant metastasis and more frequent postoperative metastasis. A reduced overall survival was strongly associated with metastasis in patients with high RET nuclear expression tumours. Our data showed that the RET nuclear expression significantly increased not only in metastatic RCC tumours but also in patients with primary tumours, who developed metastasis, compared with patients with renal tumours, who did not develop metastasis. Patients with metastatic disease typically receive systemic treatment, which is associated with substantial toxic effects. Unless the patients present with metastatic disease, clinical observation is the standard of care after nephrectomy. Therefore, biomarkers which can accurately distinguish localised tumours with high probability of metastasis from those that will remain indolent are needed. With such biomarkers, physicians can predict the patient's prognosis and consider early systemic treatment. RET immunohistochemical staining is a simple, inexpensive, and reliable assay. Considering that localised RCC tumours are usually treated by partial or radical nephrectomy, tumour tissues are routinely available for immunohistochemical staining.

However, this study did not include a prospective and external validation. Long-time spans, not a random sample, and single-centred studies decreased the robustness of this study. RET rearrangement, genetic variants, and mutation had been found in prognostic functions of many other cancer types. Whether RET presented prognostic function in RCC should be verified in future research. Furthermore, given that all the patients in this study were Chinese, the relevance of the predictive roles should be assessed in other ethnic groups.

## 5. Conclusions

Both high RET cytoplasmic and nuclear immunostaining are independent prognostic markers for ccRCC. Patients with tumours with high RET nuclear and cytoplasmic expression were at the highest risk of progressive disease and death. High cytoplasmic RET expression retained its independent poor prognostic value in patients treated with targeted drugs, and high RET nuclear expression is associated with distant metastasis. Multiple in vivo models have been considered in existing studies. The RET receptor plays an important role in tumour growth and metastasis; hence, it should be considered as a novel therapeutic target in RCC subsets. Moreover, the RET nuclear expression is an independent predictor of ccRCC postoperative metastasis. The expression of this protein in primary renal tumours can identify patients with early-stage disease, who also exhibit a high potential to develop metastasis after surgery. Overall, these findings can provide therapeutic implications in patients who might benefit from early systemic treatment after nephrectomy.

## Figures and Tables

**Table 1:** Clinical characteristics of patients.

Features	RET cytoplasm			RET nuclear		
	Low expression (*n* = 136)	High expression (*n* = 137)	*p* value	Low expression (*n* = 144)	High expression (*n* = 129)	*p* value
Median age (years)	56 (29–81, 46–63)	52 (18–79, 45–62)		54 (18–81, 45–63)	56 (21–79, 47–65)	

Gender (male)	107 (79%)	97 (71%)	0.256^*∗*^	110 (76%)	94 (73%)	0.931^*∗*^

Median PFS months	26 (1.5–>102, 10.8–>55)	12 (1–>93, 5.8–>36)	<0.001^†^	26 (1.5–102, 9.3–>65)	13.1 (1–>93, 6–>47)	0.002^†^

Median OS months	>36 (2–>102, >25.1–>44.5)	28 (1–>93, 13.1–>47)	<0.001^†^	49 (2–102, 21.4–>65)	31.5 (1–>93, 14.2–>52)	0.007^†^

ECOG PS						
0	69	63	0.151^*∗*^	78	56	0.233^*∗*^
≥1	67	74		66	73	

T stage						
1-2	90	78	0.083^*∗*^	89	79	0.907^*∗*^
3-4	46	59		55	50	

Metastasis status						
No	59	56	0.100^*∗*^	68	47	<0.001^*∗*^
Yes	77	81		76	82	

Fuhrman grade						
1-2	87	81	0.144^*∗*^	90	78	0.208^*∗*^
3-4	49	56		54	51	

Data are median (range, IQR) or *n* (%). ^*∗*^PFS and OS were deemed as continuous variable, and Spearman's Rho test was used to study the relationships between the covariates. ^†^PFS and OS were deemed as dichotomized variable, and log-rank test was used to study the differences between the covariates.

**Table 2:** Multivariable Cox regression analyses.

Multivariable Cox regression	Association with PFS			Association with OS		
	*p* value	HR	95% CI	*p* value	HR	95% CI
(A) RET cytoplasmic continuous^*∗*^						
Fuhrman grade	0.003	1.408	1.120–1.770	0.039	1.292	1.014–1.646
ECOG PS	<0.001	1.766	1.419–2.199	<0.001	1.921	1.521–2.428
T stage	0.011	1.275	1.057–1.538	0.026	1.242	1.026–1.503
Metastasis	<0.001	2.775	1.791–4.298	<0.001	3.143	1.998–4.944
Targeted treatment	0.001	0.518	0.349–0.769	<0.001	0.407	0.270–0.613
RET continuous	<0.001	1.860	1.459–2.372	<0.001	1.666	1.306–2.125

(B) RET nuclear continuous^*∗*^						
Fuhrman grade	0.003	1.408	1.123–1.765	0.039	1.287	1.012–1.636
ECOG PS	<0.001	1.842	1.470–2.308	<0.001	1.999	1.573–2.541
T stage	0.024	1.244	1.029–1.503	0.043	1.220	1.006–1.480
Metastasis	<0.001	2.671	1.697–4.203	<0.001	3.097	1.930–4.970
Targeted treatment	0.002	0.526	0.351–0.790	<0.001	0.419	0.275–0.639
RET nuclear continuous	0.004	1.379	1.109–1.716	0.047	1.260	1.003–1.584

(C) RET cytoplasmic dichotomized^*∗*^						
RET cytoplasmic (high versus low)	0.001	1.683	1.235–2.293	0.003	1.635	1.185–2.255

(D) RET nuclear dichotomised^*∗*^						
RET nuclear (high versus low)	0.020	1.428	1.058–1.927	0.307	1.180	0.859–1.623

(E) Multivariable Cox regression (combined RET cytoplasmic and nuclear expression)^*∗*†^						
Low RET nuclear and cytoplasmic	reference					
High RET nuclear only	0.028	1.713	1.059–2.772	0.083	1.568	0.943–2.608
High RET cytoplasmic only	0.220	1.366	0.830–2.248	0.136	1.507	0.879–2.582
High RET nuclear and cytoplasmic	<0.001	2.466	1.639–3.711	<0.001	2.294	1.499–3.510

^*∗*^Covariates are not showed for the omitted space; ^†^comparisons of dichotomized variable.

HR, hazard ratio; CI, confidence interval; ECOG PS, Eastern Cooperative Oncology Group performance status.
